# A case of primary metastatic glassy cell carcinoma of the uterine cervix that responded to combined docetaxel and carboplatin^☆^

**DOI:** 10.1016/j.gynor.2013.07.004

**Published:** 2013-07-22

**Authors:** Yuji Ukita, Hiroshi Tsubamoto, Toru Kato, Shigeo Suno, Hiroaki Shibahara

**Affiliations:** a Department of Obstetrics and Gynecology, Hyogo College of Medicine, 1-1 Mukogawa-cho, Nishinomiya, Hyogo 663-8501, Japan; b Department of Obstetrics and Gynecology, Kinki Central Hospital, 3-1 Kurumazuka, Itami, Hyogo 664-8533, Japan

**Keywords:** Glassy cell carcinoma, Cervical cancer, Chemotherapy, Docetaxel, Carboplatin

## Abstract

• Glassy cell carcinoma (GCC) of the cervix is rare and aggressive.
• A few case reports have described a response to intravenous chemotherapy for this malignancy.
• This is the first report of a GCC case that responded to a combination of docetaxel and carboplatin.

## Introduction

Glassy cell carcinoma (GCC) of the uterine cervix is relatively very rare, comprising < 1% of all cervical cancers. GCC is a poorly differentiated adenosquamous carcinoma with distinctive microscopic features, including ground glass-type cytoplasm, prominent nucleoli, an absence of intercellular brides, and eosinophilic inflammatory cell infiltration of the stroma. The clinical course of GCC is aggressive, and radiation and surgery are rarely curative for patients with advanced disease. Furthermore, very little is known regarding the use of chemotherapy for this cancer. In the neodjuvant setting, a clinical response to chemotherapy was described in 4 reports from Japanese centers, 3 of which were conducted using intraarterial chemotherapy and an interventional radiological technique (Mikami et al., 2000; Nagai et al., 2008). There was also a case report of a paclitaxel and carboplatin (TC) intravenous regimen used to treat 1 patient (Takekuma et al., 2006), and 2 other case reports that described a response of recurrent GCC to TC therapy (Hirashima et al., 2001; Matsuura et al., 2001). Here we report a case of primary metastatic GCC of the uterine cervix, which responded to intravenous chemotherapy with docetaxel and carboplatin (DC).

## Case

**Fig. 1:**
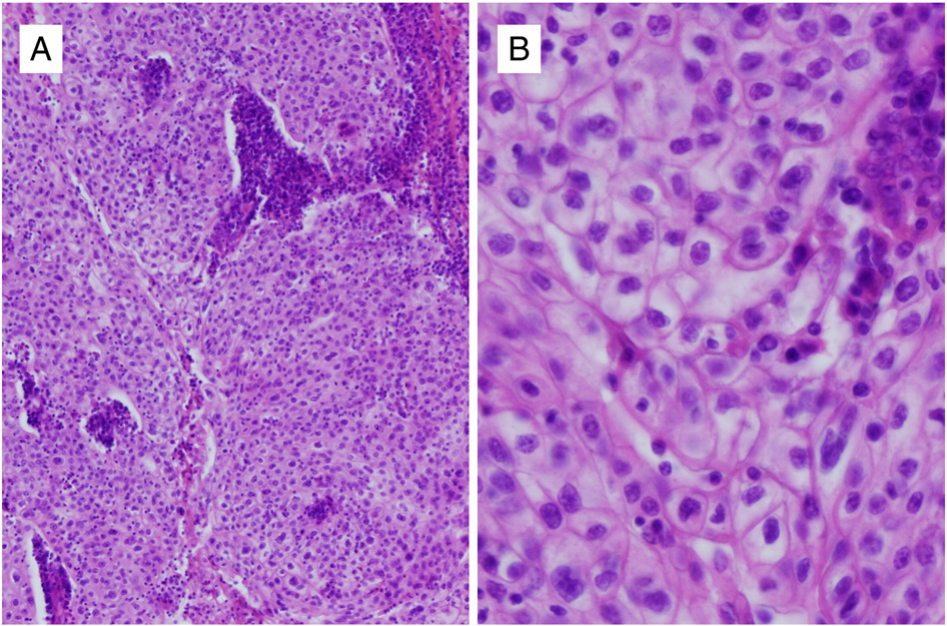
Histological diagnosis of glassy cell carcinoma (magnification: A, 40 ×; B, 400 ×).

**Fig. 2:**
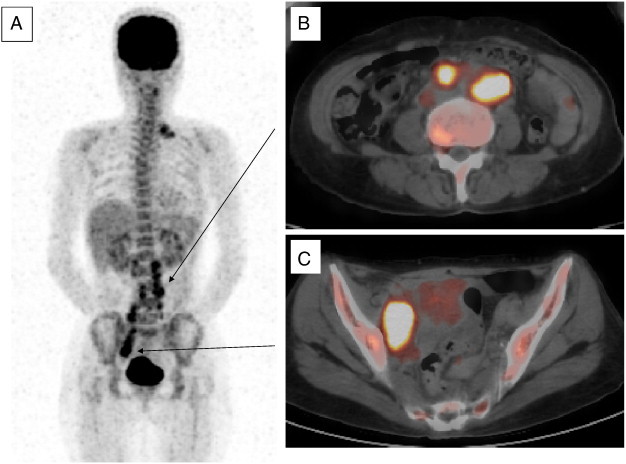
A coronal 18F-fluorodeoxyglucose (FDG)-positron emission tomography scan showing significant FDG uptake in the (A) retroperitoneal and supraclavicular lymph nodes, (B) paraaortic lymph nodes, and (C) pelvic lymph node.

A 61-year-old woman, G5P3, was referred to us because of abnormal genital bleeding. Vaginal examination revealed an exophytic tumor 5 cm in diameter originating in the cervical canal, and a normal cervix. The pedunculated tumor was excised by the same procedure as that used for the vaginal removal of prolapsed pedunculated submucous myoma. Histological examination of the specimen revealed large polygonal cells containing a distinct nucleolus and cytoplasm with a ground glass appearance. In addition, the stroma was diffusely infiltrated by lymphoplasmacytic cells. On the basis of these findings, the tumor was diagnosed as a GCC (Fig. 1A, B), and a careful examination was conducted because of the possibility of some residual cancer. Her serum CA125, SCC, and CEA levels were 3280 IU/ml, 3.8 ng/ml, and 32.4 ng/ml, respectively, and a Pap smear or cervical biopsy and curettage showed no malignancy. Magnetic resonance imaging (MRI) and 18-fludeooxyglucose positron emission tomography/computed tomography (18-FDG PET/CT) scans showed multiple retroperitoneal and supraclavicular lymph node metastases, although no residual tumor was found in the uterus (Fig. 2A–C). Therefore, systemic chemotherapy was administered, but no radical hysterectomy was performed.

**Fig. 3:**
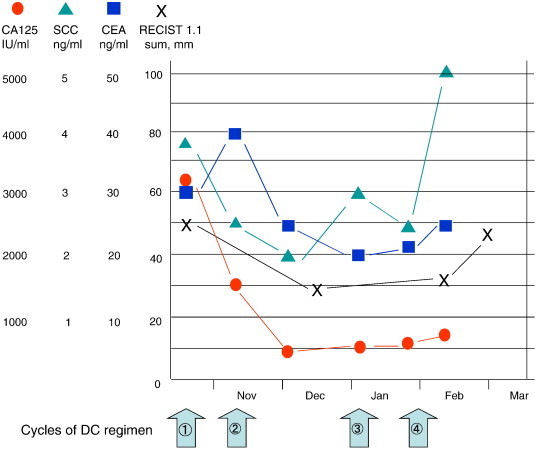
Radiological and serological response during chemotherapy consisting of docetaxel and carboplatin (DC). The duration of the response and the progression free survival were 73 days and 128 days, respectively. The radiological response was evaluated according to the Response Evaluation Criteria in Solid Tumors (RECIST) version 1.1. The sum of the diameters in the short axis of the left paraaortic and right external iliac lymph nodes is shown.

The patient was treated with DC chemotherapy (docetaxel, 60 mg/m^2^, day 1; and carboplatin, area under the curve, 6 mg·min^− 1^·mL^− 1^, day 1; both repeated every 21 days). The third cycle was delayed because of her personal circumstances. A radiological response was apparent after 2 cycles, but the disease was found to have progressed after 4 cycles. The duration of the response was therefore 73 days, and progression free survival (PFS) after DC was 128 days (Fig. 3). Over the course of the 4 treatment cycles, the patient suffered grade 4 neutropenia, grade 2 anorexia, and fatigue, as defined by the Common Terminology Criteria for Adverse Events version 4.0. As a second line therapy, she was administered irinotecan (60 mg/m^2^, days 1 and 8) and nedaplatin (a platinum analog, 80 mg/m^2^, day 1), repeated every 21 days; however, the tumor progressed over the course of the first 2 cycles. Best supportive care with a home palliative care physician was offered, and she died 315 days after the first administration of DC.

## Discussion

The standard chemotherapy for metastatic or recurrent cervical cancer is a combination of paclitaxel and cisplatin (TP). However, a recent phase III trial for primary metastatic or recurrent cervical cancer showed that TC had similar efficacy with less toxicity compared to TP (Kitagawa et al., 2012a). It should be noted though, that in the preceding phase II trial for a similar population, the same authors found that the response of adenocarcinoma/adenosquamous cell carcinoma to TC was lower than that of squamous cell carcinoma (40% v.s. 66%) (Kitagawa et al., 2012b). In another phase II trial for locally advanced or recurrent cervical cancer, Takekida et al. reported an overall response rate of 63.7%. Interestingly, in this study, the response rate was similar between non-squamous and squamous cell carcinoma (68.9% v.s. 69.7%) among chemo-naïve patients (Takekida et al., 2010). The reasons for the discrepancy in the results obtained using TC and DC remain unclear. The 3-week schedule of paclitaxel administration might impact the response of non-squamous cell cervical cancer, as demonstrated in a phase IIII trial of paclitaxel used as an adjuvant therapy for breast cancer (Sparano et al., 2008).

Based on these previous studies, we used DC to treat this patient. Although this gave an initial response, its duration was only 73 days, and the progression free survival was only 128 days. The subsequent chemotherapy consisted of irinotecan and nedaplatin. Although this has been reported to be effective for recurrent squamous cell carcinoma of the cervix (Ohara et al., 2013), it was not effective in this case. Nonetheless, this is the first report of a response DC regimen in a patient with GCC, and we suggest that its efficacy should be further investigated.

## Conflict of interest statement

The authors declare that there are no conflicts of interest.

## References

[bb0020] Hirashima Y., Kobayashi H., Nishiguchi T., Miura K., Kanayama N. (2001). A case of glassy cell carcinoma of the uterine cervix effectively responding to chemotherapy with paclitaxel and carboplatin. Anticancer Drugs.

[bb0030] Kitagawa R., Katsumata N., Shibata T., Nakanishi T., Nishimura S., Ushijima U. (2012). A randomized, phase III trial of paclitaxel plus carboplatin (TC) versus paclitaxel plus cisplatin (TP) in stage IVb, persistent or recurrent cervical cancer: Japan Clinical Oncology Group study (JCOG0505). J. Clin. Oncol..

[bb0035] Kitagawa R., Katsumata N., Ando M., Shimizu C., Fujiwara Y., Yoshikawa H. (2012). A multi-institutional phase II trial of paclitaxel and carboplatin in the treatment of advanced or recurrent cervical cancer. Gynecol. Oncol..

[bb0025] Matsuura Y., Murakami N., Nagashio E., Toki N., Kashimura M. (2001). Glassy cell carcinoma of the uterine cervix: combination chemotherapy with paclitaxel and carboplatin in recurrent tumor. J. Obstet. Gynaecol. Res..

[bb0005] Mikami M., Ezawa S., Sakaiya N., Komuro Y., Tei C., Fukuchi T., Mukai M. (2000). Response of glassy-cell carcinoma of the cervix to cisplatin, epirubicin, and mitomycin C. Lancet.

[bb0010] Nagai T., Okubo T., Sakaguchi R., Seki H., Takeda S. (2008). Glassy cell carcinoma of the uterine cervix responsive to neoadjuvant intraarterial chemotherapy. Int. J. Clin. Oncol..

[bb0050] Ohara T., Kobayashi Y., Yoshida A., Yoshioka N., Yahagi N., Kondo H. (2013). Combination of irinotecan (CPT-11) and nedaplatin (NDP) for recurrent patients with uterine cervical cancer. Int. J. Clin. Oncol..

[bb0045] Sparano J.A., Wang M., Martino S., Jones V., Perez E.A., Saphner T. (2008). Weekly paclitaxel in the adjuvant treatment of breast cancer. N. Engl. J. Med..

[bb0040] Takekida S., Fujiwara K., Nagao S., Yamaguchi S., Yoshida N., Kitada F. (2010). Phase II study of combination chemotherapy with docetaxel and carboplatin for locally advanced or recurrent cervical cancer. Int. J. Gynecol. Cancer.

[bb0015] Takekuma M., Hirashima Y., Takahashi N., Yamamichi G., Furukawa N., Yamada Y. (2006). A case of glassy cell carcinoma of the uterine cervix that responded to neoadjuvant chemotherapy with paclitaxel and carboplatin. Anticancer Drugs.

